# ​Comprehensive mendelian randomization analysis of plasma proteomics to identify new therapeutic targets for the treatment of coronary heart disease and myocardial infarction

**DOI:** 10.1186/s12967-024-05178-8

**Published:** 2024-04-30

**Authors:** Ziyi Sun, Zhangjun Yun, Jianguo Lin, Xiaoning Sun, Qingqing Wang, Jinlong Duan, Cheng Li, Xiaoxiao Zhang, Siyu Xu, Zeqi Wang, Xingjiang Xiong, Kuiwu Yao

**Affiliations:** 1 Department of Cardiovascular, Guang’anmen Hospital, China Academy of Chinese Medical Sciences, Beijing, 10053 China; 2 Graduate School, Beijing University of Chinese Medicine, Beijing, 10029 China; 3 Department of Oncology and Hematology, Dongzhimen Hospital, Beijing University of Chinese Medicine, Beijing, 10070 China; 4 Graduate School, China Academy of Chinese Medical Sciences, Beijing, 10070 China; 5 Department of Andrology, Guang’anmen Hospital, China Academy of Chinese Medical Sciences, Beijing, 10053 China; 6 Eye Hospital, China Academy of Chinese Medical Sciences, Beijing, 10040 China; 7 School of Traditional Chinese Medicine, Beijing University of Chinese Medicine, Beijing, 10070 China

**Keywords:** Coronary heart disease, Myocardial infarction, Protein, Proteome-wide mendelian randomization, Biomarker, Drug target

## Abstract

**Background:**

Ischemic heart disease is one of the leading causes of mortality worldwide, and thus calls for development of more effective therapeutic strategies. This study aimed to identify potential therapeutic targets for coronary heart disease (CHD) and myocardial infarction (MI) by investigating the causal relationship between plasma proteins and these conditions.

**Methods:**

A two-sample Mendelian randomization (MR) study was performed to evaluate more than 1600 plasma proteins for their causal associations with CHD and MI. The MR findings were further confirmed through Bayesian colocalization, Summary-data-based Mendelian Randomization (SMR), and Transcriptome-Wide Association Studies (TWAS) analyses. Further analyses, including enrichment analysis, single-cell analysis, MR analysis of cardiovascular risk factors, phenome-wide Mendelian Randomization (Phe-MR), and protein-protein interaction (PPI) network construction were conducted to verify the roles of selected causal proteins.

**Results:**

Thirteen proteins were causally associated with CHD, seven of which were also causal for MI. Among them, FES and PCSK9 were causal proteins for both diseases as determined by several analytical methods. PCSK9 was a risk factor of CHD (OR = 1.25, 95% CI: 1.13–1.38, *P* = 7.47E-06) and MI (OR = 1.36, 95% CI: 1.21–1.54, *P* = 2.30E-07), whereas FES was protective against CHD (OR = 0.68, 95% CI: 0.59–0.79, *P* = 6.40E-07) and MI (OR = 0.65, 95% CI: 0.54–0.77, *P* = 5.38E-07). Further validation through enrichment and single-cell analysis confirmed the causal effects of these proteins. Moreover, MR analysis of cardiovascular risk factors, Phe-MR, and PPI network provided insights into the potential drug development based on the proteins.

**Conclusions:**

This study investigated the causal pathways associated with CHD and MI, highlighting the protective and risk roles of FES and PCSK9, respectively. FES. Specifically, the results showed that these proteins are promising therapeutic targets for future drug development.

**Supplementary Information:**

The online version contains supplementary material available at 10.1186/s12967-024-05178-8.

## Background

Coronary heart disease (CHD) is a clinical syndrome associated with the formation of plaques in the arterial intima, leading to the narrowing and eventual occlusion of the vessels. Clinically, CHD primarily manifests as Myocardial Infarction (MI) or ischemic cardiomyopathy [1]. CHD accounts for roughly 9.1 million deaths globally each year, with a disproportionate impact on low- to middle-income regions [2]. Despite continuous progress in treatment and medical research, cardiovascular mortality rates are on the rise, and age-standardized mortality from cardiovascular disease has increased in certain regions. Furthermore, complications such as ischemia/reperfusion injury following myocardial reperfusion, in-stent restenosis, and contrast-induced nephropathy underscore the pressing need for new therapeutic targets for CHD and MI [3].

About 75% of FDA-approved drugs in 2017 targeted human proteins [4]. Proteins in the blood circulation provide essential information about human health and can be used as potential biomarkers and drug targets [5]. In contrast to tissue-specific diseases, plasma proteins have important applications in the management of cardiovascular diseases, including CHD due to their direct contact with blood vessels. Large-scale genome-wide association studies (GWAS) of plasma protein have identified over 18,000 protein quantity trait loci (pQTLs), covering more than 4,800 proteins [6]. Utilizing Mendelian Randomization (MR), we can explore the potential causal links between these potential drug target proteins and disease phenotypes, thereby develop new treatment strategies. MR functions akin to a natural randomized controlled trial, leveraging genetic variations inherited at conception to investigate the causal link between certain factors (e.g., plasma proteins) and phenotypes (e.g., coronary heart disease [CHD]). In-so-doing, it minimizes confounding biases and avoids reverse causation [7]. Utilizing genetic evidence to inform the development of drug targets can significantly enhance the likelihood of drug approval, effectively doubling the probability [8]. This underscores the value of MR as a powerful research tool in advancing our understanding and treatment of various diseases. Plasma protein-based MR strategies have identified potential therapeutic targets for diseases such as, Type 2 diabetes and stroke [5, 9]. In a recent MR study by Yang et al. [10], investigated the impact of lifestyle factors such as obesity on the development of CHD based on blood proteins. Although their study revealed important findings, additional MR studies focused on identifying plasma proteins associated with CHD and MI are advocated.

**Fig. 1:**
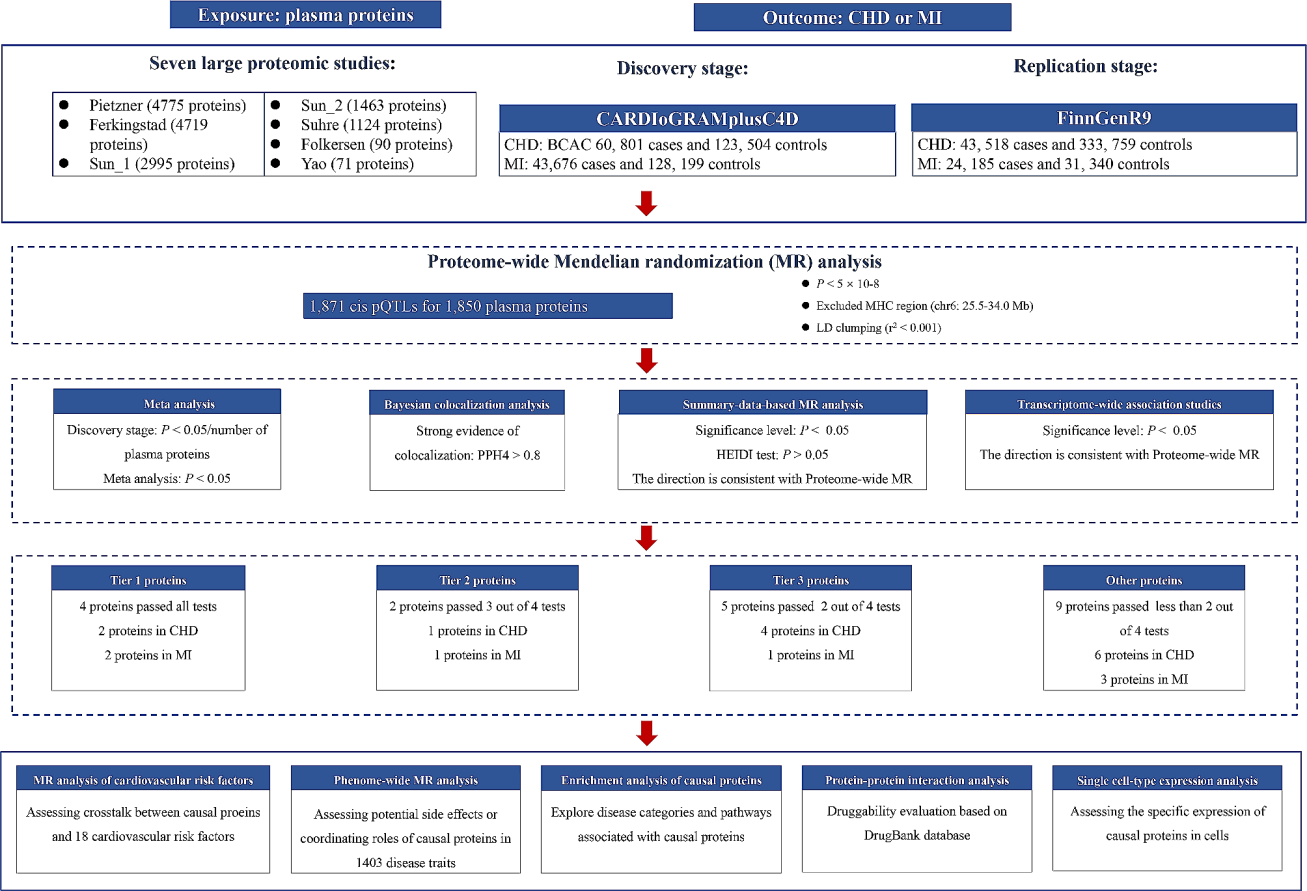
Flowchart of the study design. CHD coronary heart disease, MI myocardialinfarction

In the present study, we integrated the largest human plasma proteomic data to conduct an MR analysis to identify circulating protein markers that are causally associated with CHD and MI. To improve the reliability of the results, meta-analysis, Bayesian co-localization, Summary-data-based MR (SMR) analysis, and Transcriptome-Wide Association Study (TWAS) analysis were performed to improve the screening of proteins. The potential causal proteins were then validated at multiple levels through enrichment and single-cell analyses. The MR analysis of cardiovascular risk factors, Phenome-wide association study MR (Phe-MR), and the construction of Protein-Protein Interaction (PPI) networks were performed to provide direction for future drug development. The workflow of this study is presented in Fig. 1.

## Methods

### GWAS data for CHD and MI

The MR analysis methods was employed to identify potential therapeutic targets using CHD and MI as outcomes. In the discovery phase, GWAS summary data for CHD were derived from a Coronary Artery Disease genome-wide association meta-analysis based on the 1000 Genomes Project, conducted in 2015 by the Coronary ARtery DIsease Genome wide Replication and Meta-analysis plus The Coronary Artery Disease Genetics (CARDIoGRAM plus C4D) consortium [11]. Specifically, the study is a GWAS meta-analysis of 60,801 CHD patients and 123,504 control patients obtained from 48 studies. Most participants (77%) were from Europe, while 13% and 6% were from South Asia (India and Pakistan) and East Asia (China and Korea), respectively. CHD status was defined as MI, acute coronary syndrome, chronic stable angina, or coronary stenosis > 50%. We performed a meticulous quality control interpolation on the variants which resulted in the inclusion of 8.6 million single nucleotide polymorphisms (SNPs) and 836,000 (9%) insertions and deletions (indels). Among these, 2.7 million (29%) were categorized as low frequency variants (with a minor allele frequency [MAF] between 0.005 and 0.05). Given the gravity of MI, summary data on MI were extracted from this GWAS meta-analysis for subsequent MR analysis. The summary GWAS data for MI included 43,676 MI patients and 128,199 controls. During the replication phase, GWAS data for CHD (43,518 cases and 333,759 controls) and MI (24,185 cases and 31,340 controls) were obtained from the FinnGen R9 consortium (https://www.finngen.fi/en/access_results) [12].

### Proteomics data sources

The genetic variations of plasma proteins were selected by compiling results from seven large-scale proteomic studies (Pietzner et al. [13], 4,775 proteins; Ferkingstad et al. [14], 4,719 proteins; Sun_1 et al. [15], 2,995 proteins; Sun_2 et al. [16], 1,463 proteins; Suhre et al. [17], 1,124 proteins; Folkersen et al. [18], 90 proteins; Yao et al. [19], 71 proteins). out of which three studies lacked complete protein summary data (Pietzner et al. [13], Sun_2 et al. [16], Yao et al. [19]). A detailed description of these studies is provided in Supplementary Table S18. Given that cis-pQTLs regulate protein expression levels by directly modulating the protein transcription, translation, degradation, stability, or activity, we utilized the 1871 cis-pQTLs of 1850 proteins summarized by Sun et al. [6] from the seven proteomics studies described above as genetic tools for plasma proteins (Supplementary Table S19). Cis-pQTLs were selected based on the following criteria: (i) their significant association with proteins at the genome-wide level (*P* < 5 × 10^− 8^); (ii) SNPs and proteins were located outside of the Major Histocompatibility Complex (MHC) region (chr6: 25.5–34.0 Mb); (iii) were independently associated (linkage disequilibrium (LD) clumping r^2^ < 0.001); (iv) had the highest sum of variance explained by genetic instruments at the protein level; (v) The SNPs were located within 1 Mb of the transcription start site of the protein-coding gene. Genetic instrumentation of proteins ranged from 0.12 to 81.9% proportion of variance explained (PVE).​.

### Proteome-wide MR analysis

Initially, the strength of each genetic variant was evaluated based on the PVE and F statistics (PVE = 2 × Effect Allele Frequency (EAF) × (1-EAF) × beta2; F = PVE^2^ × (N-2)/(1- PVE ^2^)) [20]. Cis-pQTL with F-statistics greater than ten were considered strong genetic variants. Next, highly variable cis-pQTLs were extracted from GWAS of CHD and MI. These were then harmonized with protein genetic tools. Subsequently, Steiger filtering and MR analysis were conducted. In cases where the instrumental variable in MR comprised only one SNP, the Wald ratio method was employed to estimate the logarithmic change in the risk of CHD and MI per one standard deviation (SD) increase in plasma protein levels. Causality estimates for plasma proteins on CHD and MI was determined by the Inverse-Variance Weighted (IVW) methods for instrumental variables with more than one SNP. Further sensitivity analyses were performed using the Cochran Q-test and the MR-Egger intercept test to assess the heterogeneity and horizontal pleiotropy of the MR results [21, 22]. For MR analyses where the instrumental variables contain more than two SNPs, the MR results were considered robust if the weighted median, the weighted mode, the simple mode, and the MR-Egger model yield MR estimates consistent with the IVW direction. The MR-Egger result was only considered if the MR estimator was multi-directional. Steiger test was applied to explore whether there was a reverse causal relationship between exposure and outcome. Multiple test corrections were performed using the Bonferroni adjustment with a significance level of *P* divided by the number of proteins ultimately used for MR analysis. In the discovery phase, causal proteins were identified, and replication analysis was conducted using GWAS summary data from the FinnGen R9 consortium. Meta-analysis of MR estimates for both the discovery and replication phases was performed using Review Manager 5.4. When heterogeneity exceeded 50%, the IVW random effects model was utilized; otherwise, the IVW fixed effects model was applied. The significance level of meta-analysis was set at *P* < 0.05. All MR analyses were conducted using the “TwoSampleMR 0.5.8” package for R 4.3.2.

### Bayesian colocalization analysis

To investigate whether the identified causal proteins shared similar causal variants of CHD and MI within the genomic region so that interference from linkage disequilibrium is excluded, Bayesian colocalization analysis (using the coloc package in R) was performed [23]. The colocalization analysis was based on the following five hypotheses: (i) H0: No causal variant for exposure or outcome within the genomic region; (ii) H1: A single causal variant significantly associated with exposure only; (iii) H2: A single causal variant significantly associated with outcome only; (iv) H3: Existence of causal variants significantly associated with exposure or outcome within the genomic region, but driven by different causal variants; (v) H4: Exposure and outcome are driven by the same causal variant. Colocalization analyses of the identified causal proteins were performed using default parameters (p1 = 1 × 10^− 4^; p2 = 1 × 10^− 4^; p12 = 1 × 10^− 5^). Higher posterior probability for hypothesis 3 (PPH3) indicated the presence of two independent causal SNPs, each associated with a different trait [23]. In contrast, higher posterior probability for hypothesis 4 (PPH4) suggested common causal variant affecting both the exposure and outcome. In this work, PH4 > 0.8 was considered strong evidence for co-localization [23]. For proteins for which complete data were not available, complete protein data derived from the Ferkingstad [14] and Sun_3 et al. [24] studies were utilized as a proxy for the colocalization analysis. Because the same platform was employed in all studies, the comprehensive protein dataset obtained from the study by Sun_3 et al. [24] superseded that of the Sun_2 et al. [25] study. Similarly, the complete protein data from the Ferkingstad et al. [14] study was replaced by the protein data from the Pietzner et al. [13] and Sun_1 et al. [15] studies.

### SMR analysis

​Further SMR analysis was conducted to verify the causal relationship between the proteins and CHD and MI [26]. In case of more than three SNPs, the Heterogeneity in Dependent Instruments (HEIDI) test was carried out to check if the causal proteins were due to common genetic variation rather than genetic linkage. SMR analysis and HEIDI tests were conducted using SMR software (Version: 1.3.1). A significance level of *P* < 0.05 was set for the SMR analysis. A P-value > 0.05 in the HEIDI test indicated that the causal association between exposure and outcome was not influenced by linkage disequilibrium.

### TWAS analysis

​To further explore the association of these genes encoding causal proteins with CHD and MI, a TWAS analysis was conducted based on FUSION. Gene-level expression quantitative trait loci (eQTL) for whole blood were obtained from the GTEx Consortium V8 (https://gtexportal.org/). The linear sum of Z-score weights for locus-specific independent SNPs were calculated by FUSION. Subsequently, the genetic impact of CHD and MI (GWAS Z scores for CHD and MI) was combined with mRNA expression weights. Using the FUSION platform, we calculated the TWAS expression weights (i.e., SNP-gene expression correlations) based on the reference transcriptome [27]. In our analysis, we employed several prediction models, including top1, blup, lasso, enet, and bslmm. The model demonstrating the best prediction performance was chosen to calculate weights for mRNA expression. Subsequently, the estimated gene expression levels were utilized to investigate susceptibility genes associated with CHD and MI. The consistency of the TWAS Z-scores with the direction of the whole proteome MR analysis, and a *P*_TWAS_ < 0.05, were considered to be indicators of identified causal proteins passing the TWAS test at the transcriptome level.

Subsequently, we classified causal proteins into different levels of evidence based on whether they passed the tests of meta-analysis, Bayesian co-localization, SMR analysis, and TWAS analysis simultaneously. Proteins that passed all tests were categorized as tier l, those that passed three of the tests were categorized as tier ll, and proteins that passed any two tests were categorized as tier III.

### The causality from causal proteins on 18 cardiovascular risk factors

To comprehensively evaluate the influence of identified causal proteins on the cardiovascular system, we also investigated their causal connections with 18 risk factors associated with cardiovascular disease. The GWAS data of 18 cardiovascular risk factors were all derived from populations of European ancestry, comprising lipids (Total cholesterol, Triglycerides, High-density lipoprotein, Low-density Lipoprotein, Apolipoprotein A1, Apolipoprotein B, LPA), blood pressure (Systolic blood pressure, Diastolic blood pressure, Pulse pressure), Glycemic traits (Fasting glucose, Fasting insulin, two hour glucose, glycosylated hemoglobin), and Anthropometric measures (Body mass index, Waist circumference, Hip circumference, waist-to-hip ratio) (Supplementary Table S20). For causal proteins with significant associations (*P* < 0.05) with risk factors, we further investigated whether the direction of association was congruent with that observed for CHD and MI.

### Phe-MR analysis of causal proteins on 1403 disease traits

​Prior to exploring the key proteins identified through screening as potential targets for novel drug development, Phe-MR analysis was performed. This analysis predicted the potential therapeutic effects of drugs targeting these proteins on other diseases and predicts any potential adverse drug reactions. Next, we performed Phe-MR analyses using GWAS data from the UKB cohort (https://www.leelabsg.org/resources) [28], identified 28 million SNPs among 1,403 disease traits in 408,961 White British individuals (Supplementary Table S21). The disease traits were defined following the “PheCodes,” system which organizes International Classification of Diseases (ICD) codes into phenotypic results that can be used for systematic genetic analysis of multiple disease traits [28]. Results of the Phe-MR analysis method were consistent with those of the whole proteome MR analysis, demonstrating a significant causal relationship between plasma proteins and disease at *P* < 0.05/1403. The Phe-MR results are interpreted as follows: an increase in plasma protein levels per SD corresponds to either an increase or decrease in the likelihood of a specific disease or trait.

### Enrichment analysis of causal proteins and construction of PPI networks

Using a *q*-value < 0.05 as a screening condition, the causal proteins of CHD and MI were analyzed by disease ontology (DO) and the Kyoto Encyclopedia of Genes and Genomes (KEGG) enrichment using the R package clusterProfiler [29] to explore the disease categories and pathways associated with the causal proteins, respectively. Based on the ‘2023 AHA/ACC/ACCP/ASPC/NLA/PCNA Guideline for the Management of Patients With Chronic Coronary Disease’ [30] and the ‘2012 ACCF/AHA focused update of the guideline for the management of patients with unstable angina/non-ST-elevation myocardial infarction (updating the 2007 guideline and replacing the 2011 focused update): a report of the American College of Cardiology Foundation/American Heart Association Task Force on Practice Guidelines’ [31], targets were identified from the Drugbank database corresponding to drugs recommended by the CHD and MI guidelines. Subsequently, the drug-corresponding targets were merged with the causal proteins of CHD and MI. A PPI network was established using the STRING database (https://cn.string-db.org/) [32] (the minimum required interaction score was set to 0.4, and proteins with no linkage in the network would be hidden) to assess the interactions between the identified causal proteins and the drug targets.

### Validating causal proteins at the single-cell level

The expression of mRNAs corresponding to causal proteins was further explored in different cell types at the single-cell level. Human coronary plaque single-cell RNA sequencing (scRNA) data (GSE184073) [33] were downloaded from the GEO database (http://www.ncbi.nlm.nih.gov/geo/) [34] which were derived from a patient with stable angina pectoris (SAP) and a patient with MI. The Seurat 4.1.3 package in R was employed to perform Quality control, log-normalization, and identify 3000 highly variable genes. The RunPCA function was employed to conduct dimensionality reduction using principal component analysis. The ElbowPlot function establishes the appropriate principal components (PCs). The FindNeighbors function used utilized to construct a shared nearest neighbor graph for the top 30 PCs and clusters the cells using the FindClusters function with the appropriate resolution set. Annotation of cell subpopulations is based on Emoto et al. [33]. Finally, we utilized the FindAllMakers function under the criteria: ‘min. pct = 0.1, logfc. pct = 0.25, and a corrected P-value < 0.05’, to identify mRNA expression variations associated with causal proteins across different cell types, and their distribution was visualized.

## Results

### Proteome-wide MR analysis

**Fig. 2:**
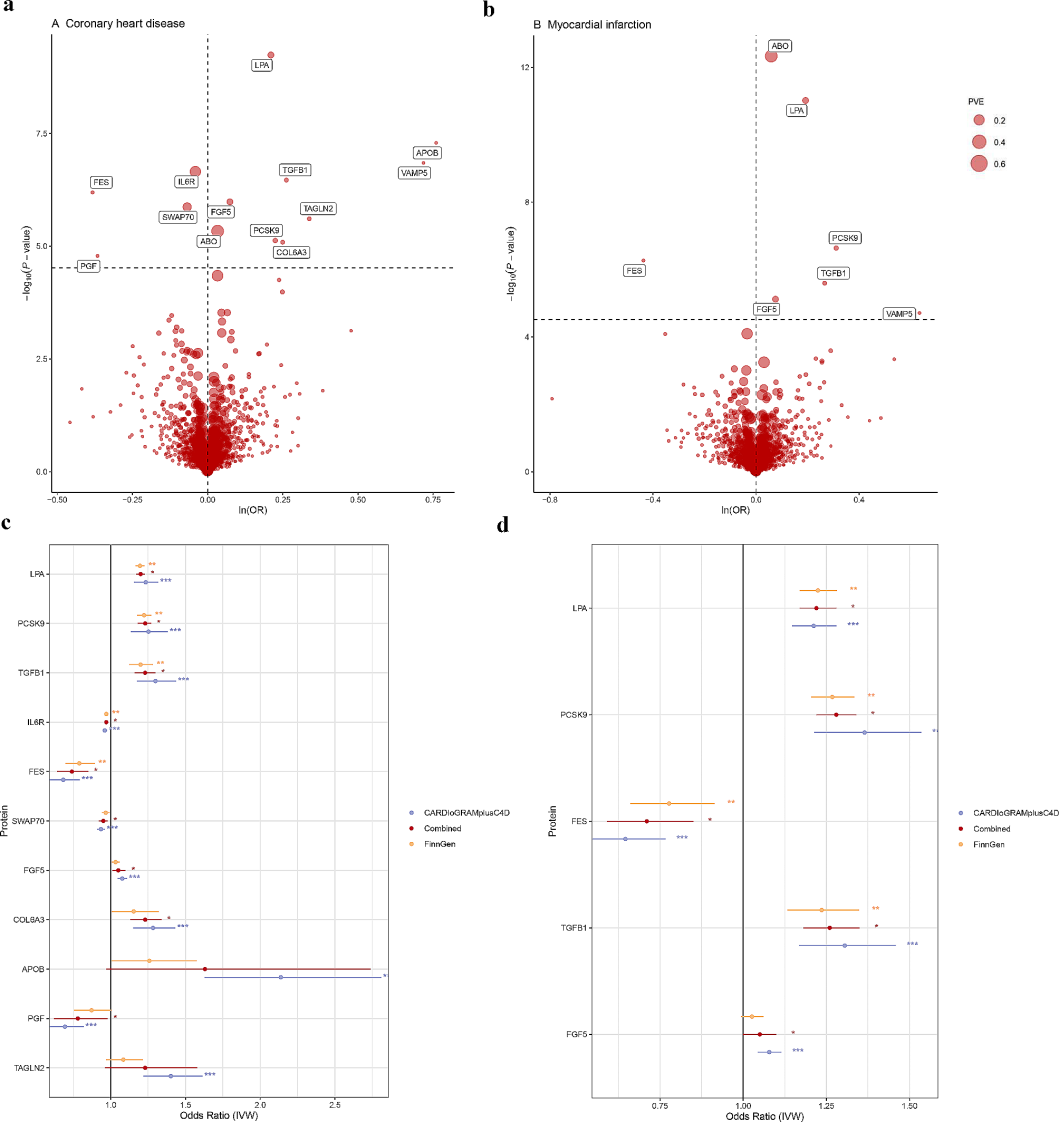
Genetically predicted circulating proteins in relation to CHD and MI risk. MR volcano plots of plasma proteins versus risk of **(a)** CHD and **(b)** MI. **(a)** and **(b)** show MR analyses of plasma proteins on CHD and MI risk using Wald ratios or IVW, respectively. OR for increased risk of CHD or MI were expressed as per SD increase in plasma protein levels. Forest plot results of circulating proteins in the discovery and replication phases in relation to **(c)** CHD and **(d)** MI risk. OR are scaled to per one SD increase in the genetically predicted circulating proteins levels. Color differences represent different data sets. *, *P* < 0.05, **, *P* < 0.01, ***, *P* < 0.001

In this study, the F-statistics of the proteins were greater than ten, indicating strong instrumental variables. The PVE by cis-pQTLs varied from 0.12 to 81.9%. In the discovery phase, 1667 plasma proteins were causally associated with CHD using either the IVW or Wald ratio method (Fig. 2a and Supplementary Table S1). Thirteen plasma proteins exhibited significant causal association with CHD (*P* < 0.05/1667). The PVE of the protein was greater than that of CHD. Furthermore, the Steiger test indicated no evidence of reverse causal association for the MR estimate. Specifically, genetically predicted increases in the levels of certain proteins, such as lipoprotein(a) (LPA) [Odds ratio (OR) = 1.23, 95% Confidence Interval (95% CI): 1.15–1.32, *P* = 5.7E-10], apolipoprotein B (APOB) (OR = 2.14, 95% CI: 1.63–2.81, *P* = 5.08E-08), vesicle associated membrane protein 5 (VAMP5) (OR = 2.05, 95% CI: 1.57–2.68, *P* = 1.42E-07), transforming growth factor beta 1 (TGFB1) (OR = 1.30, 95% CI: 1.17–1.44, *P* = 3.41E-07), fibroblast growth factor 5 (FGF5) (OR = 1.08, 95% CI: 1.05–1.11, *P* = 5.08E-08), transgelin 2 (TAGLN2) (OR = 1.40, 95% CI: 1.22–1.61, *P* = 2.46E-06), ABO (OR = 1.03, 95% CI: 1.02–1.05, *P* = 4.63E-06), and proprotein convertase subtilisin/kexin type 9 (PCSK9) (OR = 1.25, 95% CI: 1.13–1.38, *P* = 7.47E-06), snp (COL6A3) (OR = 1.28, 95% CI: 1.15–1.43, *P* = 8.15E-06) were associated with elevated risk of CHD. In contrast, higher levels of interleukin 6 receptor (IL6R) (OR = 0.96, 95% CI: 0.95–0.98, *P* = 2.21E-07), FES proto-oncogene, tyrosine kinase (FES) (OR = 0.68, 95% CI: 0.59–0.79, *P* = 6.40E-07), switching B cell complex subunit SWAP70 (SWAP70) (OR = 0.93, 95% CI: 0.91–0.96, *P* = 8.15E-06), and placental growth factor (PGF) (OR = 0.69, 95% CI: 0.59–0.82, *P* = 1.64E-05) were associated with decreased risk of CHD (Supplementary Table S2). Among them, the causal estimates for LPA and COL6A3 proteins with CHD, based on the IVW model, showed no evidence of horizontal pleiotropy (MR-Egger intercept test with *P* > 0.05). However, heterogeneity was detected in the MR estimate for LPA with CHD (Cochran Q-derived *P* = 0.003). Nevertheless, this level of heterogeneity is considered acceptable in an MR study employing a random-effects IVW model. Importantly, the 13 identified causal proteins exhibited consistent directional estimates in all MR methods (MR-Egger, Weighted median, Simple mode, Weighted mode) compared to the IVW estimates, which confirmed that our MR findings were highly reliable. The causal association of 1655 circulating proteins with MI were analyzed using MR (Fig. 2b and Supplementary Table S3). Seven plasma proteins were causally associated with MI (*P* < 0.05/1665). Moreover, it was observed that the impact of SNPs on protein levels exceeded their influence on MI risk. The Steiger test confirmed that the MR estimates were not affected by reverse causation bias. Genetically elevated levels of ABO (OR = 1.06, 95% CI: 1.04–1.08, *P* = 4.63E-13), LPA (OR = 1.21, 95% CI: 1.15–1.28, *P* = 9.60E-12), PCSK9 (OR = 1.36, 95% CI: 1.21–1.54, *P* = 2.30E-07), TGFB1 (OR = 1.31, 95% CI: 1.17–1.46, *P* = 2.54E-06), FGF5 (OR = 1.08, 95% CI: 1.04–1.11, *P* = 7.55E-06), VAMP5 (OR = 1.89, 95% CI: 1.41–2.52, *P* = 1.94E-05) were linked to an increased risk of MI, while higher levels of FES (OR = 0.65, 95% CI: 0.54–0.77, *P* = 5.38E-07) correlated with a lower risk (Supplementary Table S4). No horizontal pleiotropy was observed for LPA and MI in the IVW model (MR-Egger intercept test with *P* > 0.05). Heterogeneity in the LPA-MI causal estimate (with Cochran Q-derived *P* = 0.046) was deemed acceptable in a random-effects IVW model analysis.

During the replication and meta-analysis stages of the study, 11 CHD causal proteins were analyzed in the FinnGen cohort (Supplementary Table S5 and Fig. 2c). Meta-analysis confirmed that nine plasma proteins (LPA, IL6R, TGFB1, FES, FGF5, SWAP70, PCSK9, COL6A3, PGF) were strongly causally associated with CHD (*P*_combined < 0.05). Similarly, for MI, replication analysis in the FinnGen cohort obtained similar results for the five plasma proteins (Supplementary Table S5 and Fig. 2d). Through a meta-analysis, we confirmed the causal association of these proteins with MI (*P*_combined < 0.05).

### Bayesian colocalization analysis

Bayesian colocalization analysis demonstrated strong colocalization between APOB, TGFB1, FES, FGF5, TAGLN2, PCSK9, and CHD, indicating shared causal genetic drivers for these proteins and CHD [PPH4 >​ 0.8]. Noteworthy, the results indicated that LPA, ABO, and CHD may be driven by two causal variants in the genome [PPH3 >​ 0.8]. For MI, PCSK9, FES, TGFB1, and FGF5 were strongly colocalized with MI in the genome (PPH4 >​ 0.8). The association of ABO, LPA, and VAMP5 with MI might be influenced by two distinct causal variants (PPH3 >​ 0.8) (Supplementary Table S6 and Supplementary Figures S1 and S2).

### SMR analysis

In subsequent tests, we conduced SMR analysis and HEIDI tests to validate the results. For CHD, six out of thirteen proteins (TGFB1, FES, TAGLN2, PCSK9, COL6A3, PGF) passed both the SMR analysis and HEIDI test (*P*smr <​ 0.05 and *P*_HEIDI_ >​ 0.05). For MI, three out of seven proteins (PCSK9, FES, FGF5) passed these tests (*P*smr <​ 0.05 and *P*_HEIDI_ >​ 0.05) (Supplementary Table S7).

### TWAS analysis

After exclusion of genes without eQTL, eQTL were extracted for five genes associated with CHD (ABO, FES, IL6R, PCSK9, VAMP5) and four genes associated with MI (ABO, FES, PCSK9, VAMP5). The TWAS conducted using the FUSION method confirmed that high expression levels of the PCSK9 gene correlated with increased risk of CHD and MI. In contrast, high expression of the FES gene was associated with decreased risk (Supplementary Table S8). Similar observations were obtained in the proteome-wide MR analysis. The TWAS also revealed associations of ABO and IL6R gene with the risk of CHD whereas the VAMP5 gene was associated with the risk of MI. However, the directions of these associations were opposite to those observed in the Proteome-wide MR analysis. As a result, FES and PCSK9 passed all tests in both diseases and were categorized as Tier I. The remaining proteins implicated in disease causation were classified as Tier II or III(Supplementary Table S9 and Supplementary Fig. S13).

### Causality of potential proteins on 18 cardiovascular risk factors

**Fig. 3:**
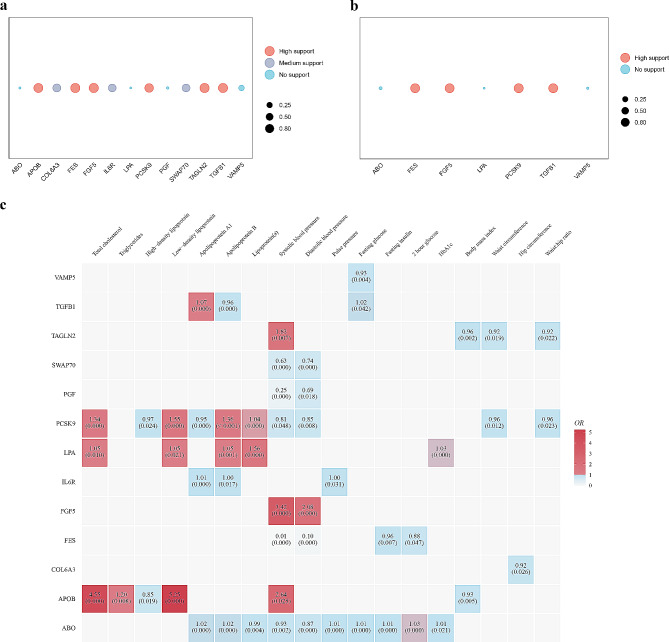
Results of colocalization analysis of **(a)** CHD causal proteins and **(b)** MI causal proteins. Circle size indicates the colocalization *P* value for PPH4, and the circle’s color indicates the evidence’s classification. **(c)** Heatmap of MR analysis of 13 causal proteins with 18 cardiovascular risk factors. In each cell, the upper value represents the OR value, and the *P* value is in parentheses

MR analysis of the 13 causal proteins associated with cardiovascular risk factors revealed that all 13 proteins were associated with one or more cardiovascular risk factors (Supplementary Table S10 and Fig. 3c). The expression levels of proteins associated with the lipid metabolism (LPA, APOB, and PCSK9) were associated with increased levels of atherosclerosis-promoting lipid indicators [Total Cholesterol, Low-density Lipoprotein (LDL)]. A decrease in PCSK9 levels correlated with increased lipids commonly considered beneficial (High-density Lipoprotein, Apolipoprotein A1). Moreover, FES exhibited protective effects on blood glucose and blood pressure, which may be one of the mechanisms by which it protects against CHD and MI.

### Phe-MR analysis of causal proteins on 1403 disease traits

Supplementary Table S11 presents the results of the Phe-MR analysis of 13 causal proteins with 1403 disease. After Bonferroni correction, ten causal proteins (ABO, APOB, FES, FGF5, IL6R, LPA, PCSK9, PGF, SWAP70, and TGFB1) were found to be significantly associated with 82 diseases or traits (*P* < 0.05/1403) (Supplementary Table S12 ). Furthermore, we specifically investigated the Phe-MR results of causal proteins related to ischemic heart disease (as a separate CHD code was not available) and MI among 1403 diseases. The findings revealed that all 12 causal proteins, except COL6A3, exhibited significant causal associations with ischemic heart disease and MI, with the directions of effect consistent with those observed in MR results (Supplementary Tables S13 and S14). This underscores the robustness of our MR findings.

**Fig. 4:**
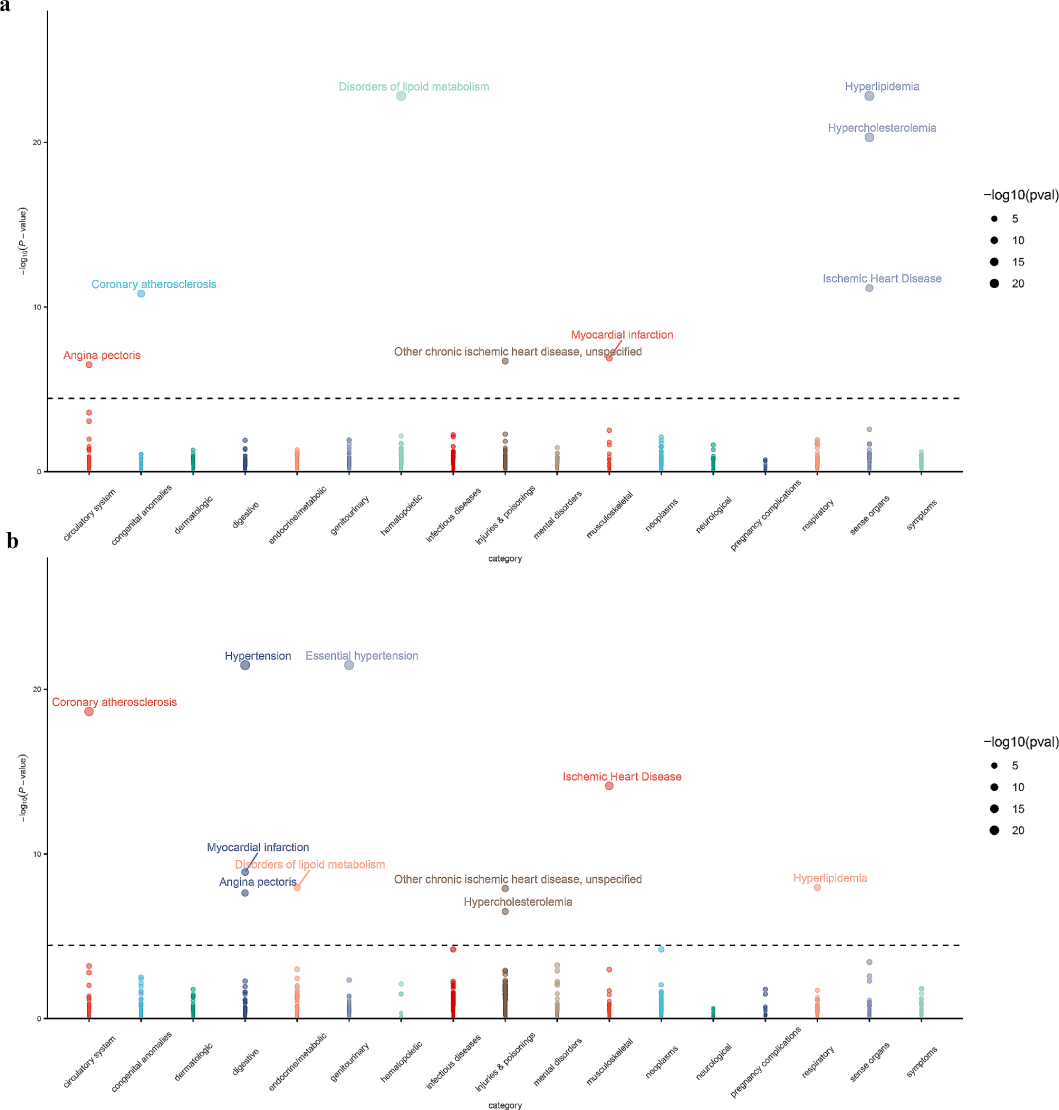
Results of Phe-MR analysis of **(a)** PCSK9 and **(b)** FES with 1403 disease traits. Horizontal coordinates represent different disease categories. The horizontal dashed line corresponds to *P* = 3.56 × 10 − 5 (0.05/1403). Diseases or characteristics statistically significant with causal proteins are labeled

​Furthermore, the Phe-MR analysis demonstrate that genetically predicted elevated PCSK9 levels were associated with an increased risk of cardiovascular diseases such as CHD and MI, as well as lipid metabolism disorders, including hyperlipidemia and hypercholesterolemia. Moreover, high levels of FES have been linked to decreased risk of CHD and MI, and other conditions such as primary hypertension and dyslipidemia (Fig. 4). The results of the Phe-MR analysis reaffirmed the MR results which showed that increased levels of PCSK9 expression were risk factors of CHD and MI, whereas FES served as a protective factor. More importantly, both PCSK9 and FES demonstrated affected the CHD-related risk factors. Given the effectiveness of PCSK9-targeted inhibitors in the treatment of CHD and lipid metabolism disorders, it is essential to explore the development of drugs targeting FES. Examining the potential of FES-targeted medications to enhance current treatments for CHD and potentially provide synergistic effects on additional risk factors is imperative.​

### Enrichment analysis of causal proteins and construction of PPI networks

**Fig. 5:**
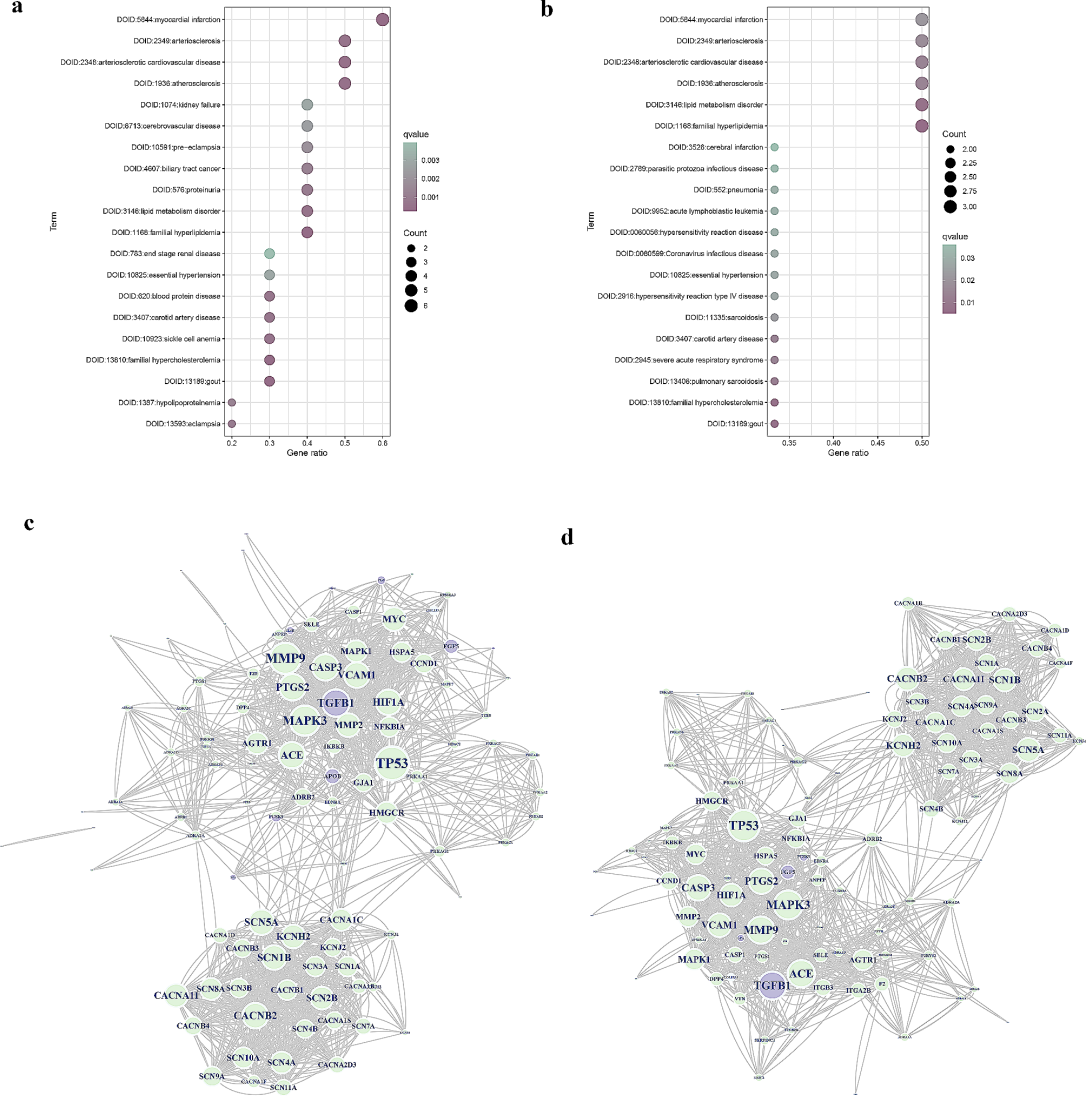
Enrichment analysis and PPI network results. Results of DO enrichment analysis for **(a)** 13 CHD causal proteins and **(b)** 7 MI causal proteins. Horizontal coordinates represent the proportion of genes corresponding to causal proteins to the total gene set. Vertical coordinates represent disease entries. **(c)** PPI network of 13 CHD causal proteins and **(d)** 7 MI causal proteins. Purple represents causal proteins, green represents corresponding drug proteins, intersecting proteins are represented by half purple and half green, and edges represent interactions between proteins

In the DO enrichment analysis, the causal proteins for CHD and MI were strongly enriched in MI, familial hyperlipidemia, familial hypercholesterolemia, atherosclerotic cardiovascular disease, and lipid metabolism disorders (Fig. 5a, b). Analysis of the KEGG enrichment analysis indicated that the causal proteins of both CHD and MI were predominantly enriched in the cholesterol metabolism pathway. In comparison, the PI3K-Akt signaling pathway showed significant enrichment in CHD-associated causal proteins, confirming the MR results (Supplementary Table S15). Ninety-eight targets of 17 drugs were predicted to treat CHD whereas 107 relevant targets for 24 drugs used for MI treatment were derived from the DrugBank database [35] (https://go.drugbank.com/) (Supplementary Table S16). To summarize the above targets, PPI networks were constructed based on the CHD and MI causal proteins. It was observed that CHD causal proteins, including TGFB1, FGF5, APOB, IL6R, PGF, PCSK9, LPA, and COL6A3, as well as four MI causal proteins, namely, TGFB1, FGF5, PCSK9, and LPA, exhibited significant interactions with existing drug targets (Fig. 5c, d). Given the effectiveness of PCSK9 inhibitors in treating CHD and MI, these findings suggest that the above causal proteins may be valid targets for new drug development.

### Validating causal proteins at the single-cell level

**Fig. 6:**
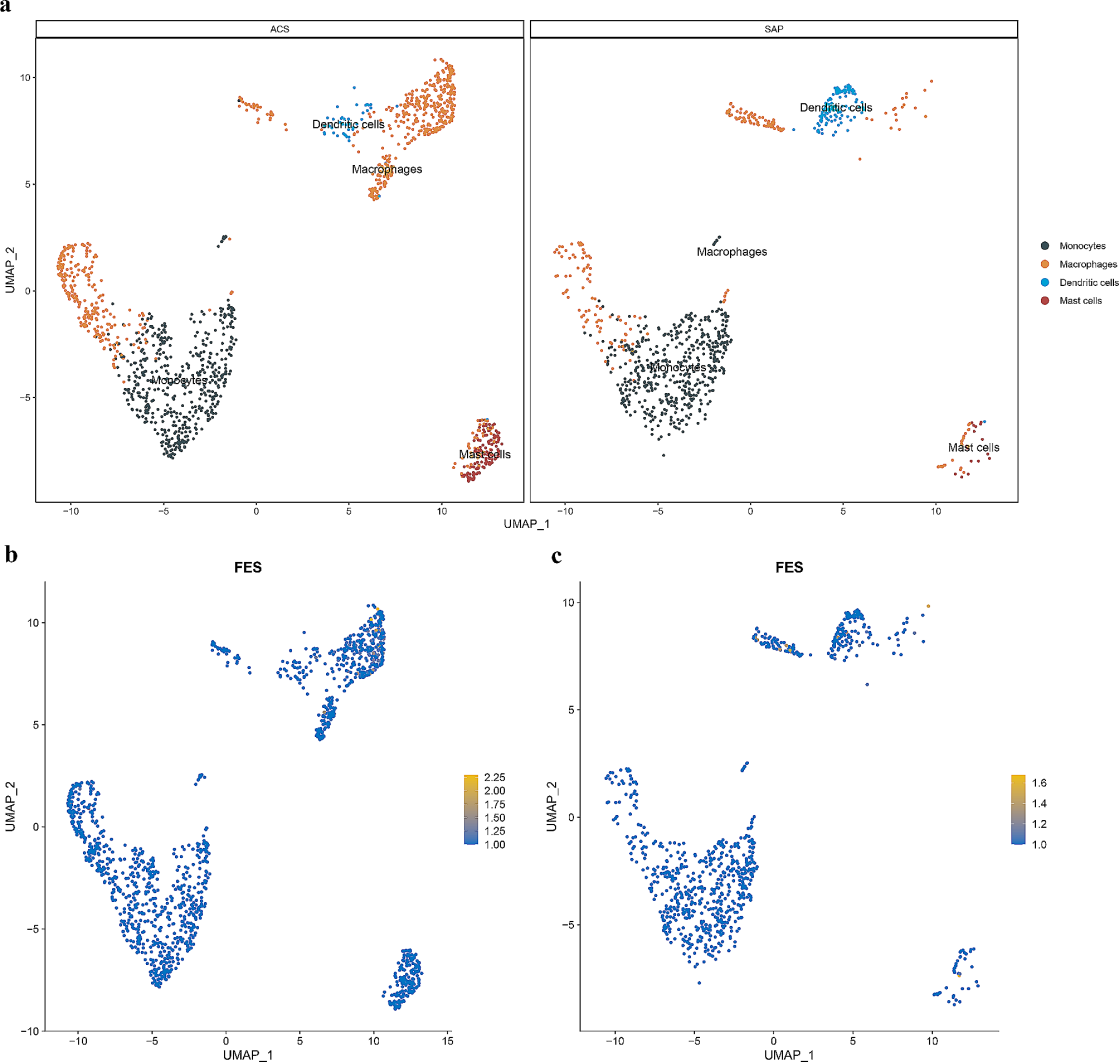
Expression of genes encoding causal proteins at the single-cell level. (**a**) Cells from MI and SAP samples were annotated into four cell subpopulations. FES gene expression in (**b**) MI and (**c**) SAP samples. The closer the color to yellow, the stronger the expression

The scRNA data were subjected to quality control, and then 2,223 cell samples (838 cell samples for SAP and 1,385 cell samples for MI) were divided into seven groups. Subsequently, seven clusters were annotated as the four cell subpopulations of monocytes, macrophages, dendritic cells, and mast cells (Fig. 6a). The expression of genes corresponding to causal proteins in different cell types was validated separately (Supplementary Figs. 4, 5). In the case of MI, the genes linked to the four causal proteins were expressed in various cell types. Specifically, FES, TGFB1, and VAMP5 were identified as being specifically expressed in macrophages (Fig. 6b). For SAP, the nine causal protein were expressed in different cell types, with FES, IL6R, and SWAP70 specifically expressed in dendritic cells (Fig. 6c and Supplementary Table S17). Because the microarray used did not include an expression assay for PCSK9, it was not possible to ascertain its expression distribution across different cell types.

## Discussions

Over the past decades, advances in pharmacologic and nonpharmacological treatments for cardiovascular disease have led to a significant reduction in cardiovascular mortality rates. However, ischemic heart disease is the leading cause of cardiovascular death worldwide [3]. Given the increasing global burden of cardiovascular disease, it is imperative to develop novel drugs for the treatment of patients. In this context, we investigated the causal relationship between 1667 and 1665 plasma proteins and the risk of CHD and MI, respectively, to identify proteins that are likely to be potential therapeutic targets through rigorous MR analysis. For CHD, proteome-wide MR analysis identified 13 protein markers, with low expression levels of four proteins and high expression levels of nine proteins associated with increased genetic susceptibility to CHD. Results of the MR analysis of MI identified seven protein markers which were associated with increased risk of MI, six of which were potential risk proteins and one was a protective protein. The protein results obtained from MR were further analyzed by integrating meta-analysis, Bayesian co-localization, SMR analysis, and TWAS analysis. Ultimately, FES and PCSK9 were the most robust causal proteins, based on these tests for both diseases. Elevated levels of PCSK9 expression were associated with increased risk of both CHD and MI, while elevated levels of FES expression were linked to decreased risk. The hierarchical relationship between CHD and MI is also reflected in their associated causal proteins, where CHD-associated causal proteins include the identified MI-associated causal proteins. Furthermore, enrichment and single-cell analyses confirmed the causal proteins from a multidimensional perspective. MR analysis of the cardiovascular risk factors, coupled with Phe-MR and PPI network construction provided crucial directions for the drug development.

This study integrates the largest human plasma proteomics data to date, with two-sample MR analysis of circulating plasma proteins to explore their causal relationships with CHD and MI. Several of the identified proteins are well-known CHD kinesins which participate in lipid metabolism processes (LPA, APOB, and PCSK9). In addition, the majority of these causal proteins were associated with CHD or MI. Yang et al. [10] found that plasma proteins such as APOB, TAGLN2, PCSK9, IL6R, and SWAP70 were causally associated with CHD through MR and co-localization analyses, which is consistent with our findings. Zheng et al. [36] combined GWAS with multi-omics data to predict causative genes associated with CHD. It was observed that 15 genes were associated with CHD, including genes that encode FES, IL6R, LPA, PCSK9, and SWAP70 proteins in this study. However, our results are more plausible because the protein data in the Yang et al. [10] study were derived from only one proteomics study, whereas in our study, we pooled protein data from seven large-scale proteomics studies. In addition, Zheng et al. [36] did not perform MR analysis; in contrast, we conducted MR analysis to identify causally associated proteins, representing a significant improvement. Furthermore, investigating the proteins directly offers more immediate implications for new drug development compared to genetic traits. Identification of COL6A3 and VAMP5 proteins in the pathogenesis of CHD and MI represents a novel study discovery, demonstrating their potential involvement in disease development.

In this study, the most compelling causal proteins for CHD and MI were PCSK9 and FES. PCSK9 is a serine protease that is synthesized in the liver and secreted into the bloodstream, where it affects cholesterol homeostasis by binding to intracellular and extracellular LDL receptors to degrade them [37]. Following the discovery that PCSK9 gain-of-function mutations were associated with familial hypercholesterolemia and loss-of-function mutations led to reduced levels of low-density lipoprotein cholesterol (LDL-C), PCSK9 rapidly became one of the most promising targets for modulating LDL-C levels [38, 39]. Inhibitors targeting PCSK9 (evolocumab, alirocumab, inclisiran) are now widely approved and recommended for the treatment of CHD [30]. In a study of 27,564 patients with stable atherosclerosis treated with statins, the combination of evolocumab significantly reduced the incidence of MI [relative risk (RR) = 0.74. 95% CI: 0.65–0.84, *P* < 0.001], stroke (RR = 0.77, 95% CI: 0.64–0.93, *P* = 0.007), and coronary revascularization (RR = 0.78, 95% CI: 0.71–0.87, *P* < 0.001) incidence during a two-year follow-up period [40]. A study involving 18,924 participants revealed that alemtuzumab contributed to a roughly 15% decrease in adverse cardiovascular events among very high-risk CHD patients with a recent diagnosis of acute coronary syndromes and persistent dyslipidemia despite receiving optimal statin therapy [41]. Notably, the FDA approved a small interfering RNA (siRNA) drug (inclisiran) targeting PCSK9 mRNA in 2021, which is designed to reduce LDL-C levels by 50% when administered once every six months. In particular, PCSK9 inhibitors have shown promising results in CHD patients experiencing side effects from prolonged statin use or who need tighter control of their LDL-C levels. Available data indicates that lipid-associated proteins, including PCSK9, are associated with increased atherosclerosis-associated lipid markers (total cholesterol, LDL-C, APOB), which is consistent with our MR analysis results on cardiovascular risk factors [42, 43]. The meta-analysis showed that PCSK9 inhibitors increased the expression of high-density lipoproteins (approximately 5.63–7.05%). However, the protective effect on lipoprotein A1 has not been confirmed through randomized controlled trials [44]. In addition, several large cohort studies have demonstrated the pheMR results of PCSK9 [39, 45]. This further verifies the reliability of our MR results, given that PCSK9 is one of the most compelling causal proteins identified in this study and that it has been successfully applied in the treatment of CHD.​.

FES belongs to the tyrosine kinase family and is involved in multiple cellular processes such as cell motility, proliferation, differentiation, survival, and inflammation [46, 47]. GWAS studies have demonstrated that the FES gene is located at the genetic locus 15q26.1, which is associated with increased risk of CHD [48]. Recent studies have indicated that the 15q26.1 locus contains at least two functional SNPs (rs17514846 and rs1894401). Both the rs17514846-A allele and the rs1894401-G allele show decreased FES expression, suggesting that the association between the 15q26.1 locus variant and CHD susceptibility may be mediated, in part, by reduced FES. Inhibition of FES by siRNA promotes the migration of monocytes and vascular smooth muscle cells, thereby accelerating atherogenesis. In addition, a CHD risk variant at the 15q26.1 locus decreased the expression of FES in monocytes and increased monocyte/macrophage abundance in atherosclerotic plaques [49]. Unlike PCSK9, which primarily regulates lipid metabolism, MR analysis of FES and cardiovascular risk factors, as well as pheMR analyzes, revealed its beneficial effects on blood glucose and blood pressure, and differences in the pathways of action may partly explain the lower connectivity of FES in the PPI network. Although evidence for the direct effect of FES on these factors is lacking, considering that the FES gene promotes neovascularization and inhibits inflammatory response, we hypothesize that this may be the mechanism by which it exerts its protective effect [46]. In addition, recent studies have shown that defects in the function of FER tyrosine kinase (FER), which belongs to the same family of nonreceptor tyrosine kinases as FES and is structurally similar, are associated with impaired mitochondrial function [50, 51]. In our study, not only were FES and PCSK9 statistically significantly and causally associated with CHD and MI, but their biological functions and involvement in pathologic process suggests their strong participation in this association. Considering the successful use of PCSK9 in CHD, this provides a possible developmental model for FES. While the precise mechanism by which FES contributes to the onset of CHD remains unclear, our study underscores its causal involvement in CHD development. In addition, FES has therapeutic potential for the control of blood glucose, blood pressure, and lipid levels, underscoring the importance of drug development targeting FES.

In addition to the key proteins mentioned above, we identified several proteins at the level of secondary evidence (FGF5, TGFB1, TAGLN2, COL6A3, PGF), which may be involved in the complex network of the disease. FGF5 can be delivered and expressed in coronary arteries to stimulate neovascularization. Administration of the FGF5 gene via the coronary arteries was reported to improve blood flow and cardiac function in patients with myocardial ischemia [52]. In addition, FGF5 stimulates hypertrophic and partial cardiomyocytes to re-enter the mitotic phase of the cell cycle, which restores the function of hibernating myocardium following MI [53]. In contrast to the cardioprotective effects of FGF5, our MR results indicate that FGF5 is a risk factor for CHD and MI although the mechanisms need to be further investigated. Elsewhere, a meta-analysis indicated that genetic variations in TGFB1 were associated with increased risk of CHD [54]. TGFB1 has been shown to promote the progression of atherosclerosis by stimulating the conversion of fibroblasts to myofibroblasts and inhibiting endothelial cell proliferation, causing endothelial mesenchymal transition [55]. Recent studies have shown that dagliflozin attenuates angiotensin II infusion-induced cardiac hypertrophy, fibrosis and increased collagen synthesis by inhibiting the TGF-β1/Smads signaling pathway [56]. TAGLN2 promotes hypoxia-induced apoptosis in cardiomyocytes [57]. Obesity and insulin resistance are important risk factors of CHD. Studies have shown that the expression of COL6A3 mRNA in adipocytes is positively correlated with body mass index and insulin resistance [58]. As a vascular endothelial growth factor family member, PGF possess bidirectional effects on CHD. On one hand, PGF facilitates myocardial neovascularization. On the other hand, it enhances the risk of plaque rupture by recruiting monocytes, improving foam cell formation, and triggering inflammatory responses [59, 60]. Consistent with our findings, an MR study conducted by Zuo et al. [61] revealed an inverse relationship between PGF levels and the risk of coronary heart disease (CHD) and myocardial infarction (MI). Furthermore, clinical evidence suggests that a rapid elevation in serum PGF levels following MI correlates positively with subsequent improvement in left ventricular function [62].

In addition, there are unavoidable limitations in this study that should be acknowledged. First, previous studies have reported that genetic variants associated with elevated risk of CHD are somewhat generalizable across ancestral groups [63]. However, the data used in this study are primarily from populations of European ancestry, and thus further confirmation is needed to improve the generalizability of these findings to other ancestries. Second, the CHD diagnosis adopted in this study contained multiple phenotypes, including chronic stable angina and acute coronary syndromes. The heterogeneity in pathophysiologic distinctions among different phenotypes suggests that the interpretation of causally of the relevant proteins need to be approached with caution. In addition, we analyzed existing open-source data; which makes it challenging to distinguish between the various phenotypes of CHD to constraints inherent in the data storage model. Although all efforts were made to explore the link between CHD and MI through side-by-side analyses, this approach limited our ability to identify specific therapeutic targets for other CHD subtypes. Future studies should also include other races and a wider range of CHD phenotypes and experimentally validate the roles and biological functions of these proteins in various diseases. Thirdly, the strict significance thresholds and criteria for grading evidence may undervalue the significance of certain proteins, including VAMP5, ABO, SWAP70, and IL6R. Even though they may not meet all the testing criteria, their association with CHD need to be further investigated. Finally, while genetic predisposition accounts for 40–60% of CHD incidence, the management of established risk factors like hypertension and smoking has been associated with reduced mortality or MI [63]. In addition, plasma proteins are influenced by various factors besides than genetics. In particular, considering the objectives of this study, future studies should aim to translate findings on proteins such as FES from our study while considering the complexity of gene-environment interactions and causal pathways between proteins and disease.​

## Conclusions

In conclusion, we conducted an MR study to systematically examine the causal relationship between plasma protein markers and the risk of CHD and MI. This study has several advantages such as the use of large sample size, comprehensive proteome coverage, multilevel bias detection, minimal risk of reverse causal associations, and extremely low confounding bias. Thirteen plasma proteins were identified to be causally associated with CHD and seven with MI. Enrichment and single-cell analyses were performed to validate these proteins. Further Phe-MR and PPI networks targeting these causal proteins revealed their potential for drug development. Given the success of PCSK9 in treating CHD, we demonstrate that FES may be a novel therapeutic target. Future work should aim to validate and develop drugs based on these potential targets.

### Electronic supplementary material

Below is the link to the electronic supplementary material.

Supplementary Material 1

Supplementary Material 2

## Data Availability

1871cis pQLTs of 1850 plasma proteins from the published article by Sun et al. (Sun 2023; 10.1186/s13073-023-01229-9IF: 12.3 Q1 B1). Summary GWAS data for coronary heart disease and myocardial infarction in the discovery stage were extracted from the IEU open GWAS project (https://gwas.mrcieu.ac.uk/). Summary GWAS data for coronary heart disease and myocardial infarction at the replication stage were available from the FinnGenR9 (https://www.finngen.fi/en/access_results). As complete GWAS data were not available for some of the proteins, complete proteins GWAS summary data from the UK Biobank Pharma Proteomics Project (Sun 2023; 10.1038/s41586-023-06592-6IF: 64.8 Q1 B1) were used in the Bayesian colocalization analysis for alternative analysis. The eQLTs for genes encoding causal proteins in Transcriptome‑wide association studies were derived from GTEx V8 (https://www.genome.gov/Funded-Programs-Projects/Genotype-Tissue-Expression-Project). All GWAS summary data for 18 cardiovascular risk factors were obtained from IEU open GWAS project (https://gwas.mrcieu.ac.uk/). Summary GWAS data for 1403 disease traits in the Phenome-wide Mendelian randomization analysis were obtained from the Lee Lab (https://www.leelabsg.org/resources). The sources of all data in the study were described in detail in the paper. All other data supporting this study’s findings are available in the Supplementary Data. Single-cell sequencing data were obtained from the Gene Expression Omnibus (GEO) of the National Center for Biotechnology Information. They can be accessed through the GEO serial accession number GSE184073. The results of this study can be obtained by contacting the corresponding author.

## Abbreviations

CHD: Coronary heart disease
MI: Myocardial infarction
MR: Mendelian randomization
SMR: Summary-data-based MR
TWAS: Transcriptome wide association studies
Phe-MR: Phenome-wide MR
PPI: Protein-protein interaction
OR: Odds ratios
GWAS: Genome-wide association studies
CARDIoGRAM: plus C4D The Coronary ARtery DIsease Genome wide Replication and Meta-analysis plus The Coronary Artery Disease Genetics
pQTLs: Protein quantitative trait loci
MHC Major: Histocompatibility Complex
LD: Linkage disequilibrium
SD: Standard deviation
EAF: Effect Allele Frequency
LDL: Lipoprotein
LPA: Lipoprotein(a)
ICD: International Classification of Diseases
DO: Disease ontology
PC: Principal components
PVE: Proportion of variance explained
IVW: Inverse variance weighted
APOB: Apolipoprotein B
VAMP5: Vesicle associated membrane protein 5
TGFB1: Transforming growth factor beta 1
FGF5: Fibroblast growth factor 5
TAGLN2: Transgelin 2
PCSK9: Proprotein convertase subtilisin/kexin type 9
COL6A3L: Collagen type VI alpha 3 chain
IL6R: Interleukin 6 receptor
FES: Proto-oncogene, tyrosine kinase
SWAP70: Switching B cell complex subunit SWAP70
PGF: Placental growth factor
SNPs: Single nucleotide polymorphisms
PPH3: Posterior probability for hypothesis 3
PPH4: Posterior probability for hypothesis 4
HEIDI: Heterogeneity in Dependent Instruments
eQTL: Expression quantitative trait loci
KEGG: Kyoto Encyclopedia of Genes and Genomes
scRNA: Single-cell RNA sequencing
SAP: Stable angina pectoris
LDL-C: Lipoprotein cholesterol
SiRNA: Small interfering RNA
RR: Relative risk
FER: FER tyrosine kinase
