# Supplementary material for: ​Comprehensive mendelian randomization analysis of plasma proteomics to identify new therapeutic targets for the treatment of coronary heart disease and myocardial infarction
Source: J Transl Med. 2024 Apr 30;22:404. doi: 10.1186/s12967-024-05178-8 (PMC11061979; doi:10.1186/s12967-024-05178-8)
Supplement: Supplementary file 1 — Supplementary Material 1 [file 12967_2024_5178_MOESM1_ESM.pdf]

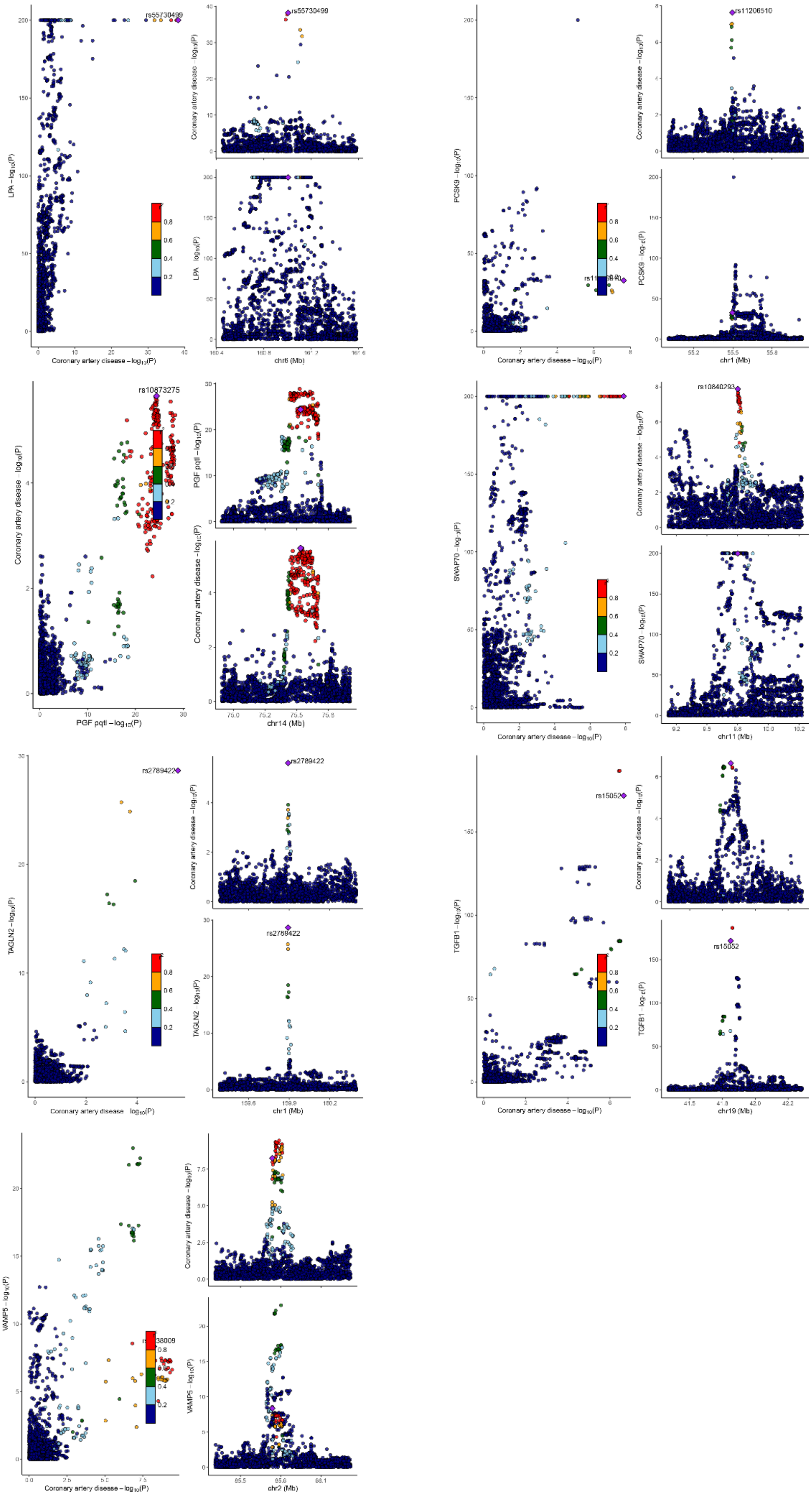

rs55730499
LPA −log10(P)
Coronary artery disease −log10(P)
chr6 (Mb)
rs11206510
PCSK9 −log10(P)
chr1 (Mb)
rs10873275
PGF pqtl −log10(P)
chr14 (Mb)
rs10840293
SWAP70 −log10(P)
chr11 (Mb)
rs2789422
TAGLN2 −log10(P)
rs15052
TGFB1 −log10(P)
chr19 (Mb)
VAMP5 −log10(P)
chr2 (Mb)
0.8
0.6
0.4
0.2

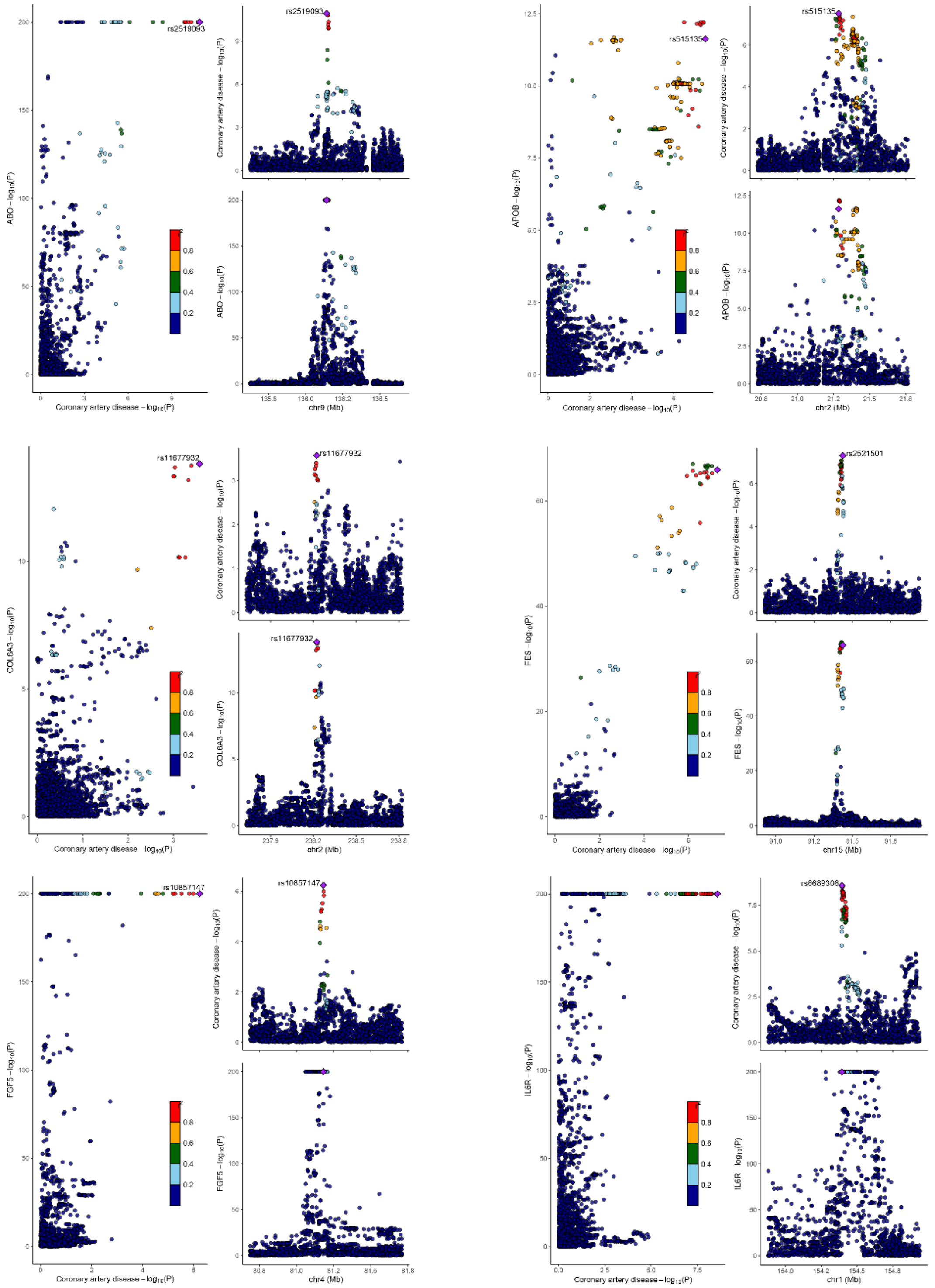

Fig S1 Regional association plot for colocalization analysis of causal protein with CHD risk. The lead SNP is shown as a purple diamond. SNPs within ±500 kb of the protein quantitative trait locus were included; p12=1e-5, prior probability a SNP is associated with both protein and CHD.

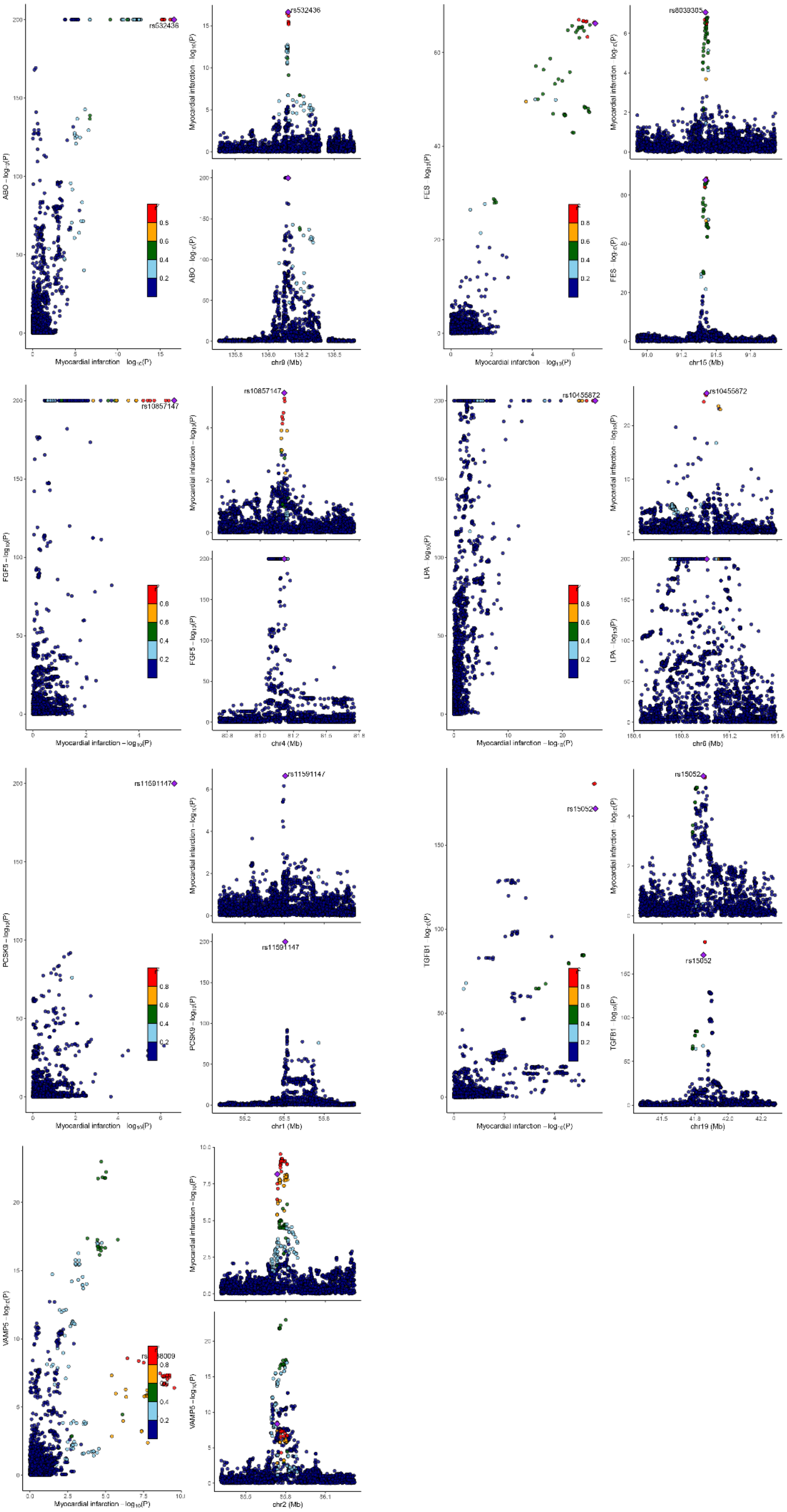

rs532436
ABO - log10(P)
Myocardial infarction - log10(P)
chr9 (Mb)
rs8038305
FES - log10(P)
chr15 (Mb)
rs10857147
FGF5 - log10(P)
chr4 (Mb)
rs10455872
LPA - log10(P)
chr6 (Mb)
rs11591147
PCSK9 - log10(P)
chr1 (Mb)
rs15052
TGFB1 - log10(P)
chr19 (Mb)
VAMP5 - log10(P)
chr2 (Mb)
0.8
0.6
0.4
0.2

Fig S2 Regional association plot for colocalization analysis of causal protein with MI risk. The lead SNP is shown as a purple diamond. SNPs within ±500 kb of the protein quantitative trait locus were included; p12=1e-5, prior probability a SNP is associated with both protein and MI.

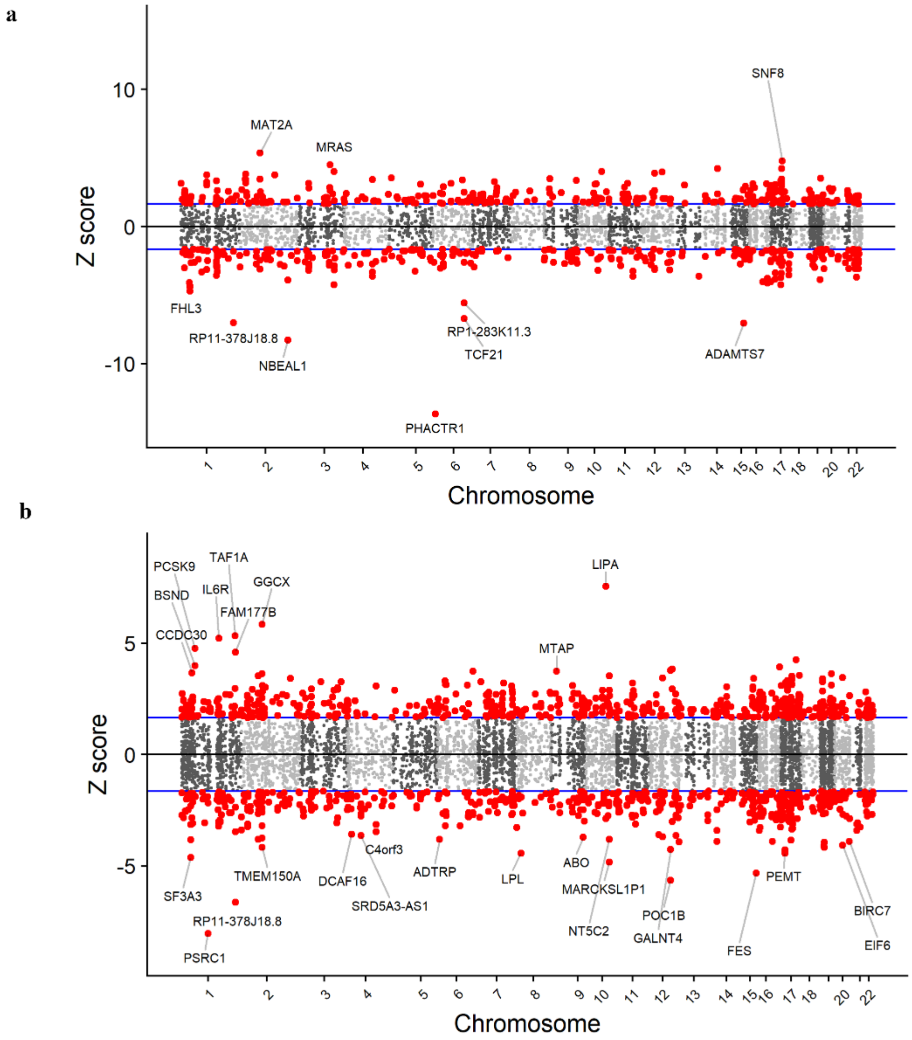

Fig S3 TWAS result of Plasma Proteins Linked to CHD and MI. (a) Results of TWAS analysis of CHD-related plasma proteins. (b) Results of TWAS analysis of MI-related plasma proteins. The horizontal axis indicates the different chromosome regions. The vertical axis is the linear sum of the Z score weights of the independent SNPs. A Z value greater than one means that there is a strong positive correlation between gene expression and disease risk.

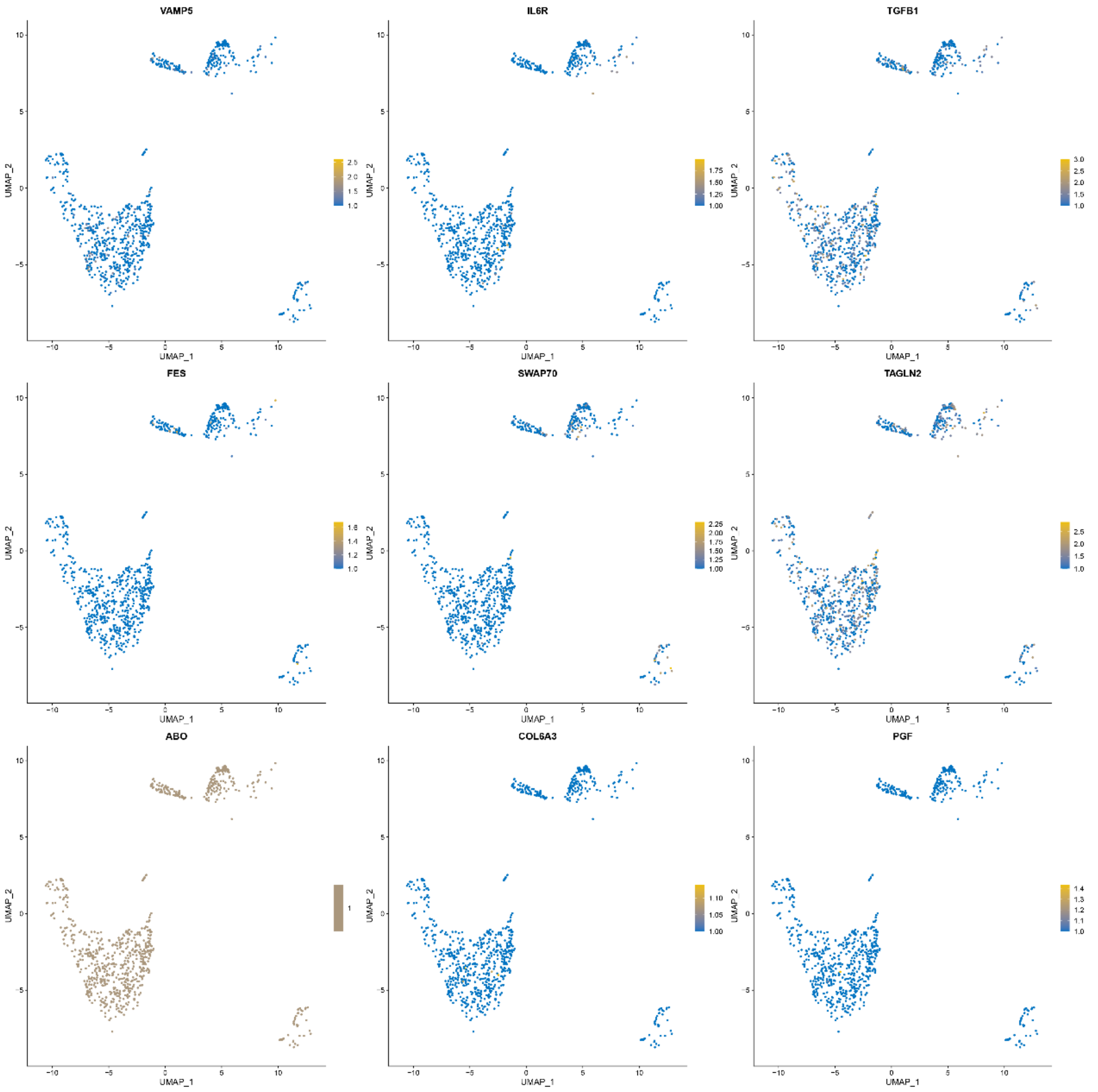

Fig S4 Expression of genes encoding CHD causal proteins at the single-cell level. The closer the color to yellow, the stronger the expression.

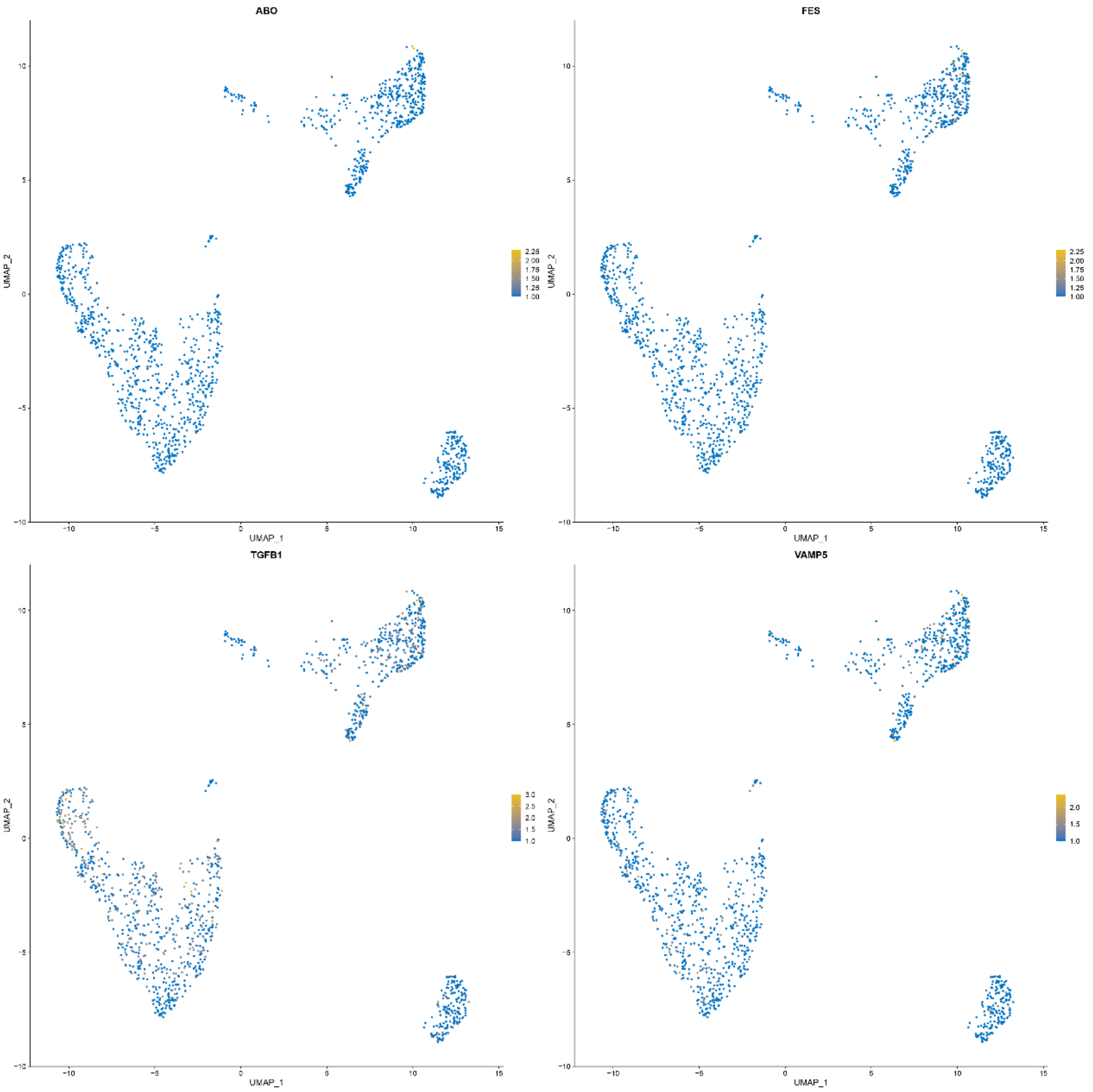

Fig S5 Expression of genes encoding MI causal proteins at the single-cell level. The closer the color to yellow, the stronger the expression.
